# Telehealth Use By Persons with Disabilities During the COVID-19 Pandemic

**DOI:** 10.5195/ijt.2021.6402

**Published:** 2021-12-16

**Authors:** Carli Friedman, Laura VanPuymbrouck

**Affiliations:** 1 CQL | The Council on Quality and Leadership, Towson, MD, USA; 2 Rush University, Chicago, IL, USA

**Keywords:** COVID-19 pandemic, Persons with disabilities, Telehealth

## Abstract

Telehealth use rapidly expanded during the COVID-19 pandemic. Understanding if, and how, persons with disabilities (PWDS) used telehealth during the pandemic is vital to assuring that this evolving and increasingly common form of health care is equitably developed and delivered to avoid reproducing the health disparities PWDS already face. Our aim was to explore the use of telehealth among PWDS during the pandemic. We conducted a weighted secondary analysis of United States Census Bureau data (April-July 2021) from 38,512 (unweighted) PWDS. Our findings revealed 39.8% of PWDS used telehealth during the second year of the pandemic, ranging from 34.5% of persons with hearing disabilities to 43.3% of persons with mobility disabilities. There were also differences in telehealth use based on sociodemographics. Telehealth promises to open doors to more equitable health care access for many PWDS, but only if access barriers are removed.

In the wake of the COVID-19 pandemic, many providers across all health care fields needed to shift to telemedicine to adhere to social-distancing guidelines and keep health care workers and patients safe. Telehealth was no longer an alternative to in-person provider visits; instead it became the only option across most of the United States (Galewitz, 2020). The Centers for Disease Control (CDC) expressed that telehealth might bridge gaps in health equity during COVID-19, especially in response to the many disparities reported among racial and ethnic communities (Chakraborty, 2021; Khanijahani, 2021). However, the CDC also acknowledged barriers to access and utilization of telehealth in many communities that could increase disparities (Centers for Disease Control and Prevention, 2020). One such community is persons with disabilities (PWDS).

There are 61 million adults with disabilities living in the United States today (Centers for Disease Control and Prevention, 2019). Despite being one of the country's largest minority communities (Brucker & Houtenville, 2015), PWDS experience significant disparities in access to health care, and the quality of care they receive can result in disparities in health and mortality (de Vries McClintock et al., 2016; Iezzoni, 2011; Kim et al., 2020; McCarthy et al., 2006; Reichard et al., 2011).

PWDS are more likely to have chronic conditions such as diabetes, heart disease, and obesity for which successful management requires regular access to quality provider support (Reichard et al., 2011). That PWDS have a thinner margin of health (Drum et al., 2005) arguably necessitates more streamlined access to equitable health care to maintain good health and functioning. PWDS also experience inequitable care due to financial, physical, attitudinal, and systemic barriers (Drainoni et al., 2006; Hwang et al., 2009; Iezzoni et al., 2021; Mudrick et al., 2012).

During the pandemic, PWDS were disproportionally affected by COVID-19 (Centers for Disease Control and Prevention, 2021a, 2021b; Chakraborty, 2021; Dobransky & Hargittai, 2020; FAIR Health et al., 2020; Turk et al., 2020). However, it is unknown how longstanding barriers to health care access contributed to these inequities, or if new barriers arose, such as related to telehealth, that also impacted person's health care access. In response to COVID-19, telehealth has been rapidly adopted as a means to provide care to maintain social distancing and isolation restrictions. Researchers have hypothesized on the probable barriers to accessing and using telehealth by persons across disability types (Annaswamy et al., 2020). For example, Americans with disabilities are less likely to have access to the internet or use of smartphones than nondisabled peers (Anderson & Perrin, 2021). Titles II and III of the Americans with Disabilities Act (ADA) requires that providers and telehealth platforms are accessible for all consumers, including those from different disability communities, and the United States Access Board has established Standards for access to effective communication (United States Department of Justice, 2014). However, there is limited enforcement and, therefore, limited compliance with these laws (Lazar & Jaeger, 2011). These may have been factors that prevented or deterred PWDS from using telehealth platforms during the coronavirus pandemic.

Though PWDS are a large social minority, there are vastly different disability experiences that produce different responses to different barriers. Moreover, many PWDS identify as having more than one disability type (Krahn et al., 2015) and this intersection might exacerbate barriers to care. Understanding if, and how, persons from across different disability communities used telehealth during the pandemic is vital to assuring that this evolving and increasingly common form of health care is equitably developed and delivered to avoid reproducing disparities in health outcomes for PWDS. Therefore, the aim of this study was to explore the use of telehealth among PWDS during the COVID-19 pandemic. To do so, we conducted a weighted secondary analysis of United States Census Bureau (2021) *COVID-19 Household Pulse Survey* data (April-July 2021) from 38,512 (unweighted) PWDS in the United States.

## METHODS

### DATA

Data were obtained from the United States Census Bureau (2021). (As it was publicly-available de-identified data, our IRB determined it was exempt from review.) To explore the impact of the COVID-19 pandemic on persons and households, the Census Bureau administered the online *COVID-19 Household Pulse Survey* to randomly selected households in the United States. Sampled households were sent text messages and emails asking them to participate. The survey, which had approximately 50 questions, covered topics such as housing, access to food, childcare, education, finances, and access to health care. Between April 14, 2021 and July 5, 2021, a total of 425,460 unduplicated persons completed the survey.

The Census Bureau asked four questions, which are used in national censuses and recommended by the United Nations, to measure disability among participants (United States Census Bureau, 2017). The disability questions included:

1. Do you have difficulty seeing, even when wearing glasses?
2. Do you have difficulty hearing, even when using a hearing aid?
3. Do you have difficulty remembering or concentrating?
4. Do you have difficulty walking or climbing stairs?

Answer options for each question are: (1) No - no difficulty; (2) Yes - some difficulty; (3) Yes - a lot of difficulty; and, (4) Cannot do at all. Persons who answer “a lot of difficulty” (3) or “cannot do at all” (4) are considered to have the disability identified in the question (yes, have the disability (1); no, do not have the disability (0) for each disability question; United States Census Bureau, 2017). In total, 38,512 unweighted participants in the sample had at least one disability; the remaining participants were removed from the sample. The Census Bureau's frequency person-weights were then applied for all analyses using SPSS27 complex samples to account for nonresponses and population demographics.

### PARTICIPANTS

Based on responses by the participants to the survey questions explained above, 17.9% had hearing disabilities, 26.9% had visual disabilities, 40.7% had mobility disabilities, and 44.2% had cognitive disabilities. Across disability types, slightly more than half of the persons in the sample were female (59.7%; Table 1). Most participants were White (77.0%), and most did not have Hispanic ethnicity (83.3%). Slightly more than half of participants (53.8%) were 54 years old or younger. Of the participants, 45.6% had a high school education or less. Almost half (45.4%) of the participants were currently married. Slightly more than one-quarter of participants (27.3%) had a total household income (pre-tax) in 2019 of less than $25,000. The most common type of health insurance was health insurance through their or their family's current or former employer (employer insurance; 48.9%), with Medicare being the second most common (36.0%). Slightly more than one-third of participants (35.7%) lived with children under 18. In terms of geographic region of the United States, 42.5% of participants lived in the South, 23.3% in the West, 19.6% in the Midwest, and 14.6% in the Northeast. Demographics differed slightly by disability type (see Table 1).

**Table 1:** Demographics of Sample

Characteristic	Subcategory	Across disability	Individual disabilities			
			Visual	Hearing	Cognitive	Mobility
Age	18 to 14	7.0%	6.0%	3.9%	11.8%	2.2%
	25 to 34	15.8%	15.0%	8.3%	25.7%	5.0%
	35 to 44	14.8%	15.2%	10.9%	20.7%	10.0%
	45 to 54	16.1%	21.1%	12.3%	16.8%	16.0%
	55 to 64	19.2%	21.2%	21.0%	13.5%	25.6%
	65 to 74	17.4%	13.5%	23.1%	7.5%	25.6%
	75+	9.6%	8.0%	20.6%	4.0%	15.5%
Sex	Male	40.3%	38.9%	54.7%	38.3%	39.9%
	Female	59.7%	61.1%	45.3%	61.7%	60.1%
Race	White alone	77.0%	68.6%	75.7%	77.1%	76.0%
	Black, alone	11.1%	14.1%	8.7%	9.6%	12.6%
	Asian, alone	3.5%	5.8%	4.2%	3.6%	2.6%
	Another race alone, or multiracial	8.4%	11.5%	11.4%	9.7%	8.8%
Ethnicity	Not Hispanic	83.3%	77.4%	81.9%	80.5%	85.0%
	Hispanic	16.7%	22.6%	18.1%	19.5%	15.0%
Education	Less than high school	4.1%	6.5%	6.8%	4.4%	5.6%
	Some high school	6.5%	7.1%	7.4%	6.4%	7.9%
	High school graduate/equivalent	35.0%	34.8%	36.9%	31.0%	39.7%
	Some college	24.0%	21.9%	20.4%	26.9%	21.2%
	Associate's degree	9.9%	9.5%	9.0%	10.3%	9.8%
	Bachelor's degree	11.9%	11.2%	10.1%	13.0%	8.7%
	Graduate degree	8.6%	9.1%	9.4%	8.0%	7.2%
Marital status	Now married	45.4%	44.2%	52.8%	40.0%	46.1%
	Widowed	7.5%	6.3%	9.9%	4.2%	12.1%
	Divorced	17.3%	19.0%	17.3%	15.0%	20.9%
	Separated	3.3%	4.5%	3.1%	3.7%	3.7%
	Never married	26.5%	26.1%	16.9%	37.1%	17.2%
Household income	Less than $25,000	27.3%	30.8%	26.7%	27.4%	32.8%
	$25,000 - $34,999	16.1%	16.3%	14.6%	14.8%	16.9%
	$35,000 - $49,999	14.5%	13.5%	15.2%	14.3%	15.0%
	$50,000 - $74,999	16.4%	14.7%	16.4%	15.8%	16.3%
	$75,000 - $99,999	10.2%	9.2%	10.3%	10.6%	8.3%
	$100,000 - $149,999	8.9%	8.0%	9.6%	9.2%	6.5%
	$150,000 - $199,999	3.4%	2.9%	3.1%	3.9%	2.0%
	$200,000+	3.2%	4.7%	4.2%	3.9%	2.2%
Health insurance/coverage	Through current/former employer of self/family (employer insurance)	48.9%	47.6%	46.1%	51.3%	41.0%
	Purchased directly from company (private insurance)	22.7%	24.6%	27.7%	18.9%	24.8%
	Medicare	36.0%	28.9%	46.5%	19.5%	54.4%
	Medicaid	30.7%	30.9%	25.2%	30.3%	36.6%
	TRICARE or other military health care	6.0%	5.0%	10.0%	5.1%	7.3%
	Veteran Affairs health care	8.8%	7.9%	14.3%	8.1%	10.3%
	Indian health service	1.8%	2.9%	3.0%	2.1%	2.2%
	Other	6.7%	8.5%	8.3%	5.9%	8.0%
Children under 18 in household	Yes	35.7%	41.6%	33.6%	42.2%	29.8%
	No	64.3%	58.4%	66.4%	57.8%	70.2%
Geographic region	Northeast	14.6%	15.6%	13.8%	14.4%	14.0%
	South	42.5%	43.2%	41.9%	41.9%	44.6%
	Midwest	19.6%	17.2%	20.3%	19.7%	20.0%
	West	23.3%	24.0%	24.0%	24.1%	21.4%

*Note.* All data are weighted. Participants could select more than one type of health insurance.

### VARIABLES AND ANALYSES

In regard to telehealth, the *Pulse Survey* asked participants: “At any time in the last 4 weeks, did you have an appointment with a doctor, nurse, or other health professional by video or by phone?” Answer options included Yes, or No. This question served as the dependent variable (DV) for this study. Sociodemographic variables (i.e., age, gender, race, ethnicity, education, marital status, income, insurance, and children in home), as well as the four disability variables described above (i.e., visual, hearing, cognitive, mobility), were used as independent variables (IVs) in this study.

In order to examine PWDS' use of telehealth during the second year of the pandemic (April-July 2021), we first conducted descriptive statistics. To explore differences in use of telehealth among persons with different disabilities, we utilized a complex samples binary logistic regression model with disability type serving as the IV, telehealth use as the DV, and demographic variables serving as the control variables (CVs). Finally, to explore correlates of telehealth access, we used complex samples binary logistic regression models with telehealth use as the DV, and sociodemographic variables serving as the IVs; separate models were conducted for across disability, as well as each of the four disability types. Confidence intervals (CI) were set at 95% for all statistical analyses.

## RESULTS

### USE OF TELEHEALTH DURING THE PANDEMIC

During the second year of the COVID-19 pandemic (April-July 2021), 39.8% of PWDS (across the four disability categories) used telehealth. Telehealth use differed by disability type with 43.3% of persons with mobility disabilities using telehealth, 42.1% of persons with cognitive disabilities, 36.8% of persons with visual disabilities, and 34.5% of persons with hearing disabilities (Table 2). Controlling for all demographics, persons with mobility disabilities were 1.28 (CI [1.13, 1.44]) times more likely to use telehealth during the pandemic than persons with other disabilities. Persons with cognitive disabilities were 1.47 (CI [1.31, 1.66]) times more likely to use telehealth during the pandemic than persons with other disabilities. Persons with hearing disabilities were 1.22 (Odds ratio (OR) = 0.82, CI [0.71, 0.94]) less likely to use telehealth during the pandemic than persons with other disabilities.

**Table 2:** Use of Telehealth during the Pandemic

Disability	Subcategory	*%* used telehealth (unadjusted)	Adjusted OR [CI]
Visual	Yes	36.8%	0.91 [0.81, 1.03]
	No	40.9%	ref
Hearing	Yes	34.5%	0.82 [0.71, 0.94]**
	No	41.0%	ref
Cognitive	Yes	42.1%	1.47 [1.31, 1.66]***
	No	38.0%	ref
Mobility	Yes	43.3%	1.28 [1.13, 1.44]***
	No	37.4%	ref

*Note*.

* *p* < 0.05.

** *p* < 0.01.

*** *p* < 0.001.

Data are weighted. OR (CI) controls for age, gender, race, Hispanic ethnicity, education, marital status, income, insurance, children in home, and geographic region.

#### ACROSS DISABILITY CATEGORIES

Across disability types, controlling for all other variables, females with disabilities were 1.16 (CI [1.05, 1.29]) more likely to use telehealth during the second year of the pandemic than males with disabilities (Table 3). PWDS from ‘another race’ or multiracial were 1.22 (CI [1.01, 1.47]) more likely to use telehealth during the pandemic than White PWDS. Compared to PWDS with graduate degrees, PWDS with less than high school (OR = 0.53, CI [0.33, 0.85]), high school degrees or equivalent (OR = 0.59, CI [0.50, 0.68]), some college (OR = 0.79, CI [0.69, 0.90]), and associate degrees (OR = 0.85, CI [0.72, 0.99]) were all less likely to use telehealth during the pandemic. Married PWDS were 1.16 (CI [1.01, 1.34]) times more likely to use telehealth during the pandemic than single PWDS. Compared PWDS who lived in the Northeast, those who lived in the South (OR = 0.70, CI [0.60, 0.82]) and Midwest (OR = 0.67, CI [0.56, 0.79]) were less likely to use telehealth.

**Table 3:** Correlates of Telehealth Use by PWDS during the Pandemic

Characteristic	Subcharacteristic	OR [CI]				
		Across disability	Individual disabilities			
			Visual	Hearing	Cognitive	Mobility
Age (ref: 18 to 24)	25 to 34	1.02 [0.77, 1.36]	1.35 [0.80, 2.29]	1.34 [0.54, 3.31]	1.04 [0.75, 1.44]	1.71 [0.74, 3.98]
	35 to 44	1.04 [0.78, 1.38]	1.59 [0.93, 2.69]	1.96 [0.80, 4.78]	1.11 [0.80, 1.54]	1.30 [0.59, 2.87]
	45 to 54	1.10 [0.83, 1.47]	2.05 [1.21, 3.48]**	2.45 [1.02, 5.87]*	1.31 [0.93, 1.83]	1.36 [0.63, 2.95]
	55 to 64	1.01 [0.76, 1.36]	1.97 [1.15, 3.35]*	2.29 [0.97, 5.41]	1.29 [0.90, 1.86]	1.20 [0.55, 2.60]
	65 to 74	0.98 [0.70, 1.37]	2.62 [1.41, 4.89]**	2.40 [0.98, 5.91]	0.99 [0.63, 1.56]	1.18 [0.53, 2.62]
	75+	0.91 [0.62, 1.33]	2.12 [1.03, 4.35]*	1.93 [0.76, 4.87]	0.91 [0.47, 1.73]	1.05 [0.46, 2.40]
Female (ref: male)		1.16 [1.05, 1.29]**	1.10 [0.91, 1.34]	0.93 [0.74, 1.17]	1.23 [1.06, 1.44]**	1.11 [0.94, 1.30]
Race (ref: White alone)	Black, alone	1.18 [1.00, 1.40]	1.04 [0.78, 1.39]	1.77 [1.16, 2.69]**	1.15 [0.89, 1.48]	1.17 [0.92, 1.50]
	Asian, alone	1.06 [0.78, 1.43]	1.12 [0.71, 1.77]	1.29 [0.69, 2.41]	0.90 [0.59, 1.39]	1.18 [0.67, 2.09]
	Another race alone, or multiracial	1.22 [1.01, 1.47]*	1.19 [0.84, 1.68]	1.26 [0.84, 1.87]	1.12 [0.87, 1.43]	1.19 [0.88, 1.62]
Ethnicity: Hispanic (ref: not Hispanic)		1.15 [0.97, 1.35]	0.90 [0.69, 1.16]	1.16 [0.81, 1.66]	1.04 [0.84, 1.30]	1.14 [0.86, 1.52]
Education (ref: graduate degree)	Less than high school	0.53 [0.33, 0.85]**	0.50 [0.25, 0.97]*	0.49 [0.23, 1.02]	0.57 [0.33, 0.99]*	0.56 [0.31, 1.00]
	Some high school	0.75 [0.55, 1.02]	1.16 [0.70, 1.91]	0.47 [0.24, 0.94]*	0.75 [0.47, 1.19]	0.79 [0.52, 1.20]
	High school graduate/ equivalent	0.59 [0.50, 0.68]***	0.57 [0.42, 0.77]***	0.64 [0.47, 0.89]**	0.50 [0.39, 0.63]***	0.65 [0.51, 0.81]***
	Some college	0.79 [0.69, 0.90]***	0.97 [0.74, 1.27]	0.95 [0.70, 1.28]	0.76 [0.62, 0.94]*	0.81 [0.66, 0.99]*
	Associate's degree	0.85 [0.72, 0.99]*	0.80 [0.59, 1.10]	0.94 [0.66, 1.35]	0.73 [0.57, 0.93]*	0.99 [0.78, 1.25]
	Bachelor's degree	0.89 [0.78, 1.02]	0.97 [0.74, 1.27]	1.14 [0.85, 1.53]	0.85 [0.69, 1.05]	0.87 [0.70, 1.08]
Marital status (ref: never married)	Now married	1.16 [1.01, 1.34]*	1.05 [0.79, 1.39]	1.10 [0.72, 1.66]	1.13 [0.93, 1.37]	1.09 [0.85, 1.39]
	Widowed	1.25 [0.97, 1.61]	1.02 [0.65, 1.60]	1.23 [0.72, 2.10]	1.05 [0.68, 1.63]	1.16 [0.82, 1.64]
	Divorced	1.08 [0.91, 1.27]	1.01 [0.72, 1.41]	1.19 [0.78, 1.84]	0.98 [0.78, 1.23]	1.02 [0.80, 1.31]
	Separated	1.14 [0.85, 1.53]	1.36 [0.82, 2.26]	0.78 [0.40, 1.54]	0.83 [0.59, 1.16]	0.98 [0.62, 1.55]
Household income (ref: $200,000+)	Less than $25,000	1.12 [0.82, 1.53]	1.01 [0.57, 1.76]	1.10 [0.53, 2.28]	1.17 [0.74, 1.86]	1.05 [0.59, 1.85]
	$25,000 - $34,999	1.31 [0.95, 1.80]	1.19 [0.68, 2.08]	1.34 [0.66, 2.74]	1.38 [0.85, 2.22]	1.37 [0.77, 2.42]
	$35,000 - $49,999	1.01 [0.74, 1.38]	0.75 [0.43, 1.33]	0.76 [0.37, 1.57]	1.04 [0.66, 1.64]	1.15 [0.65, 2.04]
	$50,000 - $74,999	1.06 [0.79, 1.42]	0.87 [0.51, 1.50]	1.06 [0.52, 2.14]	1.06 [0.68, 1.64]	1.17 [0.66, 2.06]
	$75,000 - $99,999	1.01 [0.74, 1.37]	0.96 [0.56, 1.67]	1.07 [0.52, 2.17]	1.08 [0.68, 1.73]	0.94 [0.53, 1.66]
	$100,000 - $149,999	1.02 [0.76, 1.38]	0.92 [0.54, 1.58]	0.92 [0.46, 1.84]	1.02 [0.65, 1.57]	1.16 [0.65, 2.09]
	$150,000 - $199,999	0.96 [0.66, 1.38]	0.77 [0.39, 1.49]	1.51 [0.69, 3.34]	0.98 [0.56, 1.71]	0.87 [0.46, 1.66]
Health insurance	Employer insurance (ref: no)	1.41 [1.26, 1.58]***	1.53 [1.24, 1.89]***	1.14 [0.90, 1.44]	1.50 [1.26, 1.79]***	1.30 [1.09, 1.56]**
	Private insurance (ref: no)	1.21 [1.07, 1.37]**	1.50 [1.20, 1.89]***	1.64 [1.26, 2.12]***	1.13 [0.92, 1.38]	1.10 [0.91, 1.34]
	Medicare (ref: no)	1.34 [1.16, 1.56]***	1.13 [0.85, 1.51]	1.38 [1.01, 1.89]*	1.34 [1.07, 1.68]*	1.44 [1.17, 1.77]***
	Medicaid (ref: no)	1.79 [1.57, 2.04]***	2.18 [1.72, 2.76]***	1.76 [1.32, 2.35]***	1.92 [1.59, 2.31]***	1.59 [1.31, 1.93]***
	TRICARE or other military health care (ref: no)	1.07 [0.82, 1.39]	2.08 [1.20, 3.61]**	1.11 [0.70, 1.78]	1.40 [0.98, 2.01]	0.66 [0.45, 0.97]*
	Veteran Affairs health care (ref: no)	1.68 [1.31, 2.15]***	1.09 [0.62, 1.92]	1.86 [1.22, 2.85]**	1.46 [1.02, 2.10]*	2.26 [1.61, 3.15]***
	Indian health service (ref: no)	0.85 [0.52, 1.38]	0.27 [0.11, 0.65]**	0.47 [0.17, 1.28]	0.64 [0.34, 1.22]	1.05 [0.51, 2.17]
	Other (ref: no)	1.06 [0.86, 1.33]	1.71 [1.15, 2.54]**	1.35 [0.86, 2.13]	0.99 [0.70, 1.40]	0.91 [0.66, 1.26]
Children under 18 in household (ref: no)		0.98 [0.87, 1.11]	0.97 [0.79, 1.19]	1.20 [0.92, 1.57]	0.90 [0.76, 1.05]	1.15 [0.95, 1.40]
Geographic region (ref: Northeast)	South	0.70 [0.60, 0.82]***	0.63 [0.47, 0.85]**	0.83 [0.58, 1.18]	0.74 [0.58, 0.94]*	0.73 [0.56, 0.94]*
	Midwest	0.67 [0.56, 0.79]***	0.52 [0.38, 0.72]***	0.92 [0.64, 1.34]	0.71 [0.55, 0.90]**	0.75 [0.58, 0.98]*
	West	0.93 [0.79, 1.10]	0.75 [0.55, 1.02]	1.07 [0.74, 1.56]	0.94 [0.74, 1.21]	1.21 [0.93, 1.59]

*Note*.

* *p* < 0.05.

** *p* < 0.01.

*** *p* < 0.001.

All data are weighted.

PWDS with employer insurance were 1.41 (CI [1.26, 1.58]) times more likely to use telehealth during the pandemic than those not insured with employer insurance. PWDS with private insurance were 1.21 (CI [1.07, 1.37]) times more likely to use telehealth during the pandemic than persons who did not have private insurance. PWDS who were Medicare beneficiaries were 1.34 (CI [1.16, 1.56]) times more likely to use telehealth during the pandemic than persons who were not Medicare beneficiaries. PWDS who were Medicaid beneficiaries were 1.79 (CI [1.57, 2.04]) times more likely to use telehealth during the pandemic than persons who were not Medicaid beneficiaries. PWDS who were insured/covered through Veteran Affairs (VA) were 1.68 (CI [1.31, 2.15]) times more likely to use telehealth during the pandemic than persons who were not insured through the VA.

#### PERSONS WITH VISUAL DISABILITIES

Specific to persons with visual disabilities, controlling for all other variables, persons with visual disabilities aged 18-24 were less likely to use telehealth during the pandemic than persons with visual disabilities aged 45 and greater (ORs ranged from 1.97 to 2.62). Compared to persons with visual disabilities with graduate degrees, persons with visual disabilities with less than high school (OR = 0.50, CI [0.25, 0.97]), and high school degrees or equivalent (OR = 0.57, CI [0.42, 0.77]) were less likely to use telehealth during the pandemic. Persons with visual disabilities who lived in the South (OR = 0.63, CI [0.47, 0.85]) and Midwest (OR = 0.52, CI [0.38, 0.72]) were less likely to use telehealth than persons with visual disabilities who lived in the Northeast.

Persons with visual disabilities with employer insurance were 1.53 (CI [1.24, 1.89]) times more likely to use telehealth during the pandemic than persons with visual disabilities without employer insurance. Persons with visual disabilities with private insurance were 1.50 (CI [1.20, 1.89]) times more likely to use telehealth during the pandemic than persons with visual disabilities who did not have private insurance. Persons with visual disabilities who were Medicaid beneficiaries were 2.18 (CI [1.72, 2.76]) times more likely to use telehealth during the pandemic than persons with visual disabilities who were not Medicaid beneficiaries. Persons with visual disabilities who were insured through TRICARE or other military health care were 2.08 (CI [1.20, 3.61]) times more likely to use telehealth during the pandemic than persons with visual disabilities who were not insured through TRICARE/military health care. Persons with visual disabilities who were covered by the Indian Health Service were 3.70 (OR = 0.27, CI [0.11, 0.65]) times less likely to use telehealth during the pandemic than persons with visual disabilities who were not covered by the Indian Health Service. Persons with visual disabilities who were insured through ‘other’ forms of insurance were 1.71 (CI [1.15, 2.54]) times more likely to use telehealth during the pandemic than persons with visual disabilities who were not insured through ‘other’ forms of insurance.

#### PERSONS WITH HEARING DISABILITIES

Controlling for all other variables, persons with hearing disabilities aged 45-54 were 2.45 (CI [1.02, 5.87]) times more likely to use telehealth during the pandemic than persons with hearing disabilities aged 18-24. Black persons with hearing disabilities were 1.77 (CI [1.16, 2.69]) times more likely to use telehealth during the pandemic than White persons with hearing disabilities. Compared to persons with hearing disabilities with graduate degrees, persons with hearing disabilities with some high school were 2.13 (OR = 0.47, CI [0.24-0.94]) times less likely to use telehealth during the pandemic, and persons with hearing disabilities with high school degrees were 1.56 (OR = 0.64, CI [0.47, 0.89]) times less likely.

Persons with hearing disabilities with private insurance were 1.64 (CI [1.26, 2.12]) times more likely to use telehealth during the pandemic than persons with hearing disabilities without private insurance. Persons with hearing disabilities who were Medicare beneficiaries were 1.38 (CI [1.01, 1.89]) times more likely to use telehealth during the pandemic than persons with hearing disabilities who were not Medicare beneficiaries. Persons with visual disabilities who were Medicaid beneficiaries were 1.76 (CI [1.32, 2.35]) times more likely to use telehealth during the pandemic than persons with hearing disabilities who were not Medicaid beneficiaries. Persons with hearing disabilities who were insured through VA were 1.86 (CI [1.22, 2.85]) times more likely to use telehealth during the pandemic than persons with hearing disabilities who were not insured through the VA.

#### PERSONS WITH COGNITIVE DISABILITIES

Controlling for all other variables, females with cognitive disabilities were 1.23 (CI [1.06, 1.44]) times more likely to use telehealth during the pandemic than males with cognitive disabilities. Compared to persons with cognitive disabilities with graduate degrees, persons with cognitive disabilities with less than high school education (OR = 0.57, CI [0.33, 0.99]), high school degrees (OR = 0.50, CI [0.39, 0.63]), some college (OR = 0.76, CI [0.62, 0.94]), and associate's degrees (OR = 0.73, CI [0.57, 0.93]) were all less likely to use telehealth during the pandemic. Compared to persons with cognitive disabilities who lived in the Northeast, those who lived in the South (OR = 0.74, CI [0.58, 0.94]) and Midwest (OR = 0.71, CI [0.55, 0.90]) were less likely to use telehealth.

Persons with cognitive disabilities with employer insurance were 1.50 (CI [1.26, 1.79]) times more likely to use telehealth during the pandemic than those persons with cognitive disabilities without employer insurance. Persons with cognitive disabilities who were Medicare beneficiaries were 1.34 (CI [1.07, 1.68]) times more likely to use telehealth during the pandemic than persons with cognitive disabilities who were not Medicare beneficiaries. Persons with cognitive disabilities who were Medicaid beneficiaries were 1.92 (CI [1.59, 2.31]) times more likely to use telehealth during the pandemic than persons with cognitive disabilities who were not Medicaid beneficiaries. Persons with cognitive disabilities who were insured through the VA were 1.46 (CI [1.02, 2.10]) times more likely to use telehealth during the pandemic than persons with cognitive disabilities who were not insured through the VA.

#### PERSONS WITH MOBILITY DISABILITIES

Controlling for all other variables, compared to persons with mobility disabilities with graduate degrees, persons with mobility disabilities with high school degrees (OR = 0.65, CI [0.51, 0.81]), and some college (OR = 0.81, CI [0.66, 0.99]), were all less likely to use telehealth during the pandemic. Persons with mobility disabilities who lived in the South (OR = 0.73, CI [0.56, 0.94]) and Midwest (OR = 0.75, CI [0.58, 0.98]) were less likely to use telehealth than persons with mobility disabilities who lived in the Northeast.

Persons with mobility disabilities with employer insurance were 1.30 (CI [1.09, 1.56]) times more likely to use telehealth during the pandemic than those persons with mobility disabilities without employer insurance. Persons with mobility disabilities who were Medicare beneficiaries were 1.44 (CI [1.17, 1.77]) times more likely to use telehealth during the pandemic than persons with mobility disabilities who were not Medicare beneficiaries. Persons with mobility disabilities who were Medicaid beneficiaries were 1.59 (CI [1.31, 1.93]) times more likely to use telehealth during the pandemic than persons with mobility disabilities who were not Medicaid beneficiaries. Persons with mobility disabilities who were insured through TRICARE or other military health care were 1.50 (OR = 0.66, CI [0.45-0.97]) times less likely to use telehealth during the pandemic than persons with mobility disabilities who were not insured through TRICARE/military health care. Persons with mobility disabilities who were insured through the VA were 2.26 (CI [1.61, 3.15]) times more likely to use telehealth during the pandemic than persons with mobility disabilities who were not insured through the VA.

## DISCUSSION

The aim of this study was to explore the use of telehealth among PWDS during the second year of the COVID-19 pandemic (April-July 2021). Our findings showed 39.8% of PWDS used telehealth within the last month of the survey during the second year of the pandemic. Telehealth proponents make broad claims its use increases access to care for persons in rural communities, that are typically underserved or vulnerable, and to individuals who are unable to connect with providers for any number of reasons (Dinesen et al., 2016). The results of this study indicate that for some members of the disability community this assertion is likely true, but for many others it is not. Given the significant proportion of a PWDS accessing telehealth during the pandemic in our study, it is important to ensure telehealth is accessible for this community as the pandemic continues and beyond. Understanding the use of telehealth by persons from different disability groups may expose those members from within the larger group of at the most risk of inequitable access issues and, ultimately, disparate health outcomes.

### DIFFERENCES IN USE OF TELEHEALTH BY DISABILITY TYPE

The findings of this study suggest that persons with mobility disabilities were significantly more likely to use telehealth during the second year of the pandemic compared to persons with other disabilities. Other research indicates that for persons with mobility impairments virtual health care is feasible, and liked by the majority of users (Crotty et al., 2014; Robb et al., 2019; Sechrist et al., 2018). Persons with mobility disabilities' use of telehealth may be in large part due to the well documented transportation barriers to health care access faced by this population (Wolfe et al., 2020). During the pandemic, persons with mobility limitations who would traditionally use paratransit or other rideshare methods to access medical care reported concerns of exposing themselves to COVID-19 making telehealth a safer alternative (Cochran, 2020); moreover, many public transportation systems reduced their schedules and routes during the pandemic due to smaller ridership (Kim, 2021).

In contrast, persons with hearing disabilities were significantly less likely to use telehealth during the second year of the pandemic. In general, there is a lack of universal design and a failure to adopt web accessibility standards in telemedicine platforms (Annaswamy et al., 2020). The lack of sign language, captions, and/or knowledge of providers of the need to include and how to use these features likely also create barriers for persons who have hearing disabilities (Jesus et al., 2021).

Given telehealth may be inaccessible for persons with certain intellectual and developmental disabilities (IDD) and other cognitive impairments (Krysta et al., 2021; Young & Edwards, 2020), this study's findings that persons with cognitive disabilities were more likely to use telehealth than persons without cognitive disabilities was unanticipated. While we posit this may be in part due to how cognitive disability was defined by the Census Bureau - difficulty remembering or concentrating -there may also be differences in telehealth access among persons with different types of cognitive disabilities, such as persons with IDD, traumatic brain injury, dementia, psychiatric disabilities, etc. that we were unable to explore. For example, there has been a growing use of online and virtual platforms to support health management of individuals with ‘severe mental illness’ (SMI; Ben-Zeev et al., 2014; Naslund et al., 2015). While telehealth does include challenges to providing effective treatment for persons with SMI, the pandemic exacerbated anxiety and other mental health concerns (Miu et al., 2020). In fact, Miu et al. (2020) found a significantly higher number of telehealth visits for persons with SMI during the pandemic compared to those without SMI. Dementia is most often experienced by older adults and within this community the COVID-19 pandemic had strict social isolation restrictions recommended due to the risks associated with mortality from COVID-19 (Bonanad et al., 2020). Community-dwelling older adults with dementia may have turned to telehealth as a means to access health care without the risk of exposure. In fact, a recent literature review found rural patients with mild dementia and their caregivers were highly satisfied with their telehealth visits and all would use it again (Sekhon et al., 2020). In addition, many persons with IDD were forced to isolate during the pandemic not only because they lived in congregate settings that required them to stay home, but also because of the increased threat COVID-19 represented to them (Bradley, 2020; Embregts et al., 2020); as such, they may have been more likely to use telehealth if it was available to them. Staff or others may have supported persons with IDD to use telehealth to make up for a lack of accessibility (Valdez et al., 2021).

### DIFFERENCES IN TELEHEALTH ACCESS BASED ON OTHER SOCIODEMOGRAPHIC FACTORS

In addition to differences across disability types, telehealth access is also an education issue - persons with less education were less likely to use telehealth during the pandemic no matter what type of disability they had. In fact, research has found similar educational disparities in telehealth utilization in the general public in the United States (Fischer et al., 2020). The demands of using telehealth require knowledge and/or experience on how to navigate complex health care systems as well as technology (Triana et al., 2020). In fact, Jesus et al. (2021) describe how the need for signed consent in order to utilize telehealth platforms may be a primary barrier for telehealth use by individuals with low literacy levels. Especially problematic is that research exploring feasibility and satisfaction of telehealth have reported limited recruitment of participants with lower educational levels (Bove et al., 2018; Robb et al., 2019), which could further increase barriers for persons with less education if their voices are neglected in telehealth development.

Age was a nonfactor in telehealth usage across disability types, despite concerns of a technological divide that reduces the likelihood of telehealth use in adults over the age of 65 in the general public (Fischer et al., 2020). There were very few differences in telehealth use across age groups in our study of PWDS, except for among persons with visual disabilities where persons older than 44 were more likely to use telehealth than 18-24-year-olds. Older adults, including those with disabilities, are more likely to have chronic health conditions such as diabetes, heart conditions, or arthritis that necessitate more frequent and regular health care visits to manage these conditions well (Centers for Disease Control and Prevention, 2013). However, it is difficult to conjecture why there were not more differences based on age within the disability community, especially given age-based technological divides in the general population.

We also found a number of differences in PWDS' telehealth use based on their health insurance/coverage. As expected, PWDS with employer and private insurance were more likely to use telehealth than persons without employer/private insurance. However, less expected was that PWDS covered by Medicare and/or Medicaid were more likely to use telehealth than those not covered by Medicare and/or Medicaid. The combination of expanded federal Medicare and Medicaid coverage and reimbursement for telehealth is likely the reason (Centers for Medicare and Medicaid, 2020b). Additionally, many states expanded Medicaid coverage to include a greater range of telehealth services including non-physician services, such as occupational and physical therapy, as well as other services previously denied (Centers for Medicare and Medicaid, 2020a; Nouri et al., 2020).

However, some disparities based on health insurance/coverage were notable. Persons with visual disabilities covered by the Indian Health Service were 4 times *less* likely to be able to access telehealth than persons with visual disabilities with other forms of insurance/coverage. Individuals living in rural communities or on Tribal lands are less likely to have access to highspeed internet (Bauerly et al., 2019). In fact, a study of households in the Navajo Nation, found the majority of households report not having broadband Internet service (Graves et al., 2020). Additionally, the Indian Health Service is chronically underfunded by the federal government and frequently lacks telehealth technology/availability (Bernard et al., 2017). Attempts must be made to strengthen the infrastructure of the Indian Health Service, including the availability of telehealth by the Indian Health Service and for Indigenous persons, including those with disabilities.

In addition, there were conflicting levels of access to telehealth for persons covered by TRICARE/military health care and VA health care. Across disability, persons covered by VA were more likely to use telehealth; so too were persons with hearing, cognitive, and mobility disabilities covered by VA. While persons with visual disabilities covered by TRICARE/other military health care were also more likely to use telehealth, persons with mobility disabilities with TRICARE/military health care were two times less likely to use telehealth. A recent study reported approximately two-thirds of VA patients preferred video visits versus in-person due to ease of access, quality of care, and necessity of care (Slightam et al., 2020). A more recent report showed that by June of 2020, 58% of veteran care was being provided virtually, with the majority of these being mental health or primary health care encounters (Ferguson et al., 2021). Yet, this same report found individuals aged 45 and older were less likely to use telehealth, showing some generational gap in acceptance among this population (Ferguson et al., 2021). The inconsistencies in the findings from Slightam et al. (2020) and Ferguson et al. (2021), coupled with this study's findings demonstrate the need for closer analysis of telehealth access and use by active military and veterans with disabilities.

## LIMITATIONS

When interpreting the findings from this study, a number of limitations should be noted. This study only explored real-time synchronous interactions via telehealth by PWDS during the pandemic in the United States. This was a secondary data analysis and, as such, we did not have the ability to ask follow-up questions or add additional variables. For example, we do not have information on how many PWDS tried to access telehealth but were unable to do so. We also did not have information about access to telehealth prior to the pandemic. There are likely other types of disabilities that were not captured by the Census Bureau's questions about disabilities. Finally, it is unclear what steps the Census Bureau took to make the *Pulse* survey accessible for PWDS, especially those with cognitive disabilities.

## IMPLICATIONS

There has been substantial advancement in telehealth, including evidence of the effectiveness of telehealth, in the last decade prior to COVID-19 (Darkins et al., 2008; Dinesen et al., 2016). With the COVID-19 pandemic, telehealth grew exponentially and became many persons' primary means to access health care. This study's findings suggest that, despite differences in telehealth use across disability categories and sociodemographics, telehealth is a promising form of health care for the disability community. One potential reason for this is the key policy changes that occurred across many health insurance types, and across state and federal programs to expand telehealth access; we believe this expansion should remain post-pandemic.

However, for PWDS, this new form of health care access may exacerbate current barriers and generate unforeseen barriers. Disparities in Internet accessibility and technology access and a lack of inaccessibility in telehealth services and platforms have highlighted how technology developers, academia, clinicians, and governments have failed to consider the telehealth needs of PWDS and telehealth's usability for this population (Dobransky & Hargittai, 2016; Lazar & Jaeger, 2011). Under Titles II and III of the ADA health care organizations, and state and federal programs are required to make all services accessible, including telehealth, and not exclude PWDS (Annaswamy et al., 2020). However, poor accountability by provider groups and health organizations and lack of enforceability may further the inequities already recognized and experienced by PWDS, including related to telehealth access (Powers et al., 2017; Walter-McCabe, 2020). We believe this study's findings can be used to critically appraise these barriers to begin the work to eliminate them.

The disparities unearthed in our study regarding use of telehealth highlight the need for technology developers, medical and health care organizations, and individual providers to recognize not only the unique needs of the disability community at large, but also different needs among varying disability groups. Accessibility features of telehealth need to be greatly expanded in order to ensure equitable access for all members of society, including users who are Deaf, blind, and/or have IDD. This should include incorporation of screen readers, speech recognition, closed-captioning, layout and ease of navigation, and that low literacy and technology literacy should be considered during development and design. The latter recommendation in particular attends to our finding that across disability groups there was a lack of telehealth use based on educational level. Moreover, to ensure optimal usability and acceptability of these features by PWDS, persons with a wide range of disabilities should be included as team members or consultants in the development, testing, and implementation of telehealth platforms.

## CONCLUSION

PWDS are frequently an overlooked population in regards to public health research and policy development (Krahn et al., 2015). The failure to recognize this community and their needs likely contributes to why PWDS were disproportionally impacted by COVID-19. Telehealth promises to open doors to more equitable health care access for many PWDS, but this can only occur if significant attempts are made to reduce disparities in telehealth and technology, and to expand the accessibility of telehealth technology and practices for PWDS.
